# Motor Performance Before, During and After COVID-19 and the Role of Socioeconomic Background: A 10-Year Cohort Study of 68,996 Third Grade Children

**DOI:** 10.1186/s40798-025-00968-w

**Published:** 2026-01-07

**Authors:** Robert Stojan, Katharina Utesch, Ludwig Piesch, Malte Jetzke, Jochen Zinner, Dirk Büsch, Till Utesch

**Affiliations:** 1 Martin Luther University Halle-Wittenberg, Halle (Saale), Germany; 2 University of Muenster, Muenster, Germany; 3 German Aerospace Center, Hamburg, Germany; 4 University of Luebeck, Luebeck, Germany; 5 German University of Health and Sport, Berlin, Germany; 6 Carl von Ossietzky University of Oldenburg, Oldenburg, Germany

**Keywords:** Motor performance, COVID-19, Physical fitness, Children, Socioeconomic factors, Exercise test, Longitudinal studies, Educational status, Physical education and training, Pandemic

## Abstract

**Background:**

In response to the COVID-19 pandemic, various measures—including restrictions on children’s physical activities, such as national lockdowns (LD)—were implemented to contain its spread. These measures may have compromised motor development, particularly among children from lower socioeconomic backgrounds (SEBs), who are typically less active than peers from higher SEBs. This study examined the impact of COVID-19 restrictions on motor development in relation to SEB.

**Methods:**

Data from 68,996 children in Germany (Age: 8.83 ± 0.56 years, range: 6.4–13.0; 35,270 female, 51.1%) assessed between 2011/2012 and 2022/2023 were analyzed from the longitudinal study ‘Berlin hat Talent’. Assessments before and after the pandemic used the German Motor Fitness Test, covering endurance, strength, coordination, and flexibility. Demographic data were collected via questionnaires; SEB was derived from official school-type classifications. Linear mixed-effect models accounted for hierarchical data: test values (level 1), motor domains (2a), participants (2b), and schools (3b). Motor performance was expressed as z-scores based on German reference percentiles. Effects of Time (pre, post LD I, post LD II), Motor Domain, and SEB (continuous, -2 to 2) were estimated, controlling for Age , Gender, and Secular Trends.

**Results:**

The effect of Time was significant (*p* = .014, η^2^ < .01), with motor performance lower after LD II than pre-pandemic. Time × Motor Domain interaction showed motor domain-specific changes (*p* = .001, η^2^ < .01): endurance improved, while strength, coordination, and flexibility declined. Time × Motor Domain × SEB interaction was also significant (*p* < .001, η^2^ = .01), indicating that the effect of Time differed across motor domains depending on SEB. Adjusting for Secular Trends revealed that the pandemic’s overall impact (~ –4% across domains) was even stronger (*p* < .001, η^2^ = .29), with domain-specific changes to –15.47% to + 7.56%. The SEB gap slightly closed, as higher SEB groups declined more strongly (*p* < .001, η^2^ = .10).

**Conclusions:**

The findings indicate domain-specific and SEB-related differences in motor performance during the pandemic, in particular after accounting for secular trends. Results underscore the need for ongoing monitoring and targeted support measures, particularly for children with lower SEB, during periods of disrupted daily activity.

**Supplementary Information:**

The online version contains supplementary material available at 10.1186/s40798-025-00968-w.

## Key Points

- Motor performance trajectories in different cohorts of 68,996 third-grade children were analyzed before, during, and after the COVID-19 pandemic, considering secular trends and the moderating effects of socioeconomic background.
- Motor performance showed significant declines in coordination, strength, and flexibility during the pandemic, while endurance increased, with an average drop of 4 percentile points in overall motor performance after lockdowns; after adjusting for recent secular trends.
- Children from higher socioeconomic backgrounds showed stronger declines in motor performance, with the impact varying between different motor domains, such that coordination and endurance were more affected than strength and flexibility.

**Supplementary Information:**

The online version contains supplementary material available at 10.1186/s40798-025-00968-w.

## Introduction

In response to the COVID-19 pandemic, governments worldwide imposed unprecedented restrictions on social and physical activity [1]. In Germany, school and sport closures, limits on public gatherings, and stay-at-home policies effectively contained the virus but also reduced opportunities for movement and social interaction. These disruptions were associated with increased sedentariness, mental-health issues, and lower physical activity across populations [2–4]. While most research focused on adults, evidence suggests that children also have been affected in various ways, particularly through lower physical activity and potentially compromised motor development [5–7]. Thus, there is a growing body of research suggesting that the abrupt cessation of daily routines brought about by the pandemic and associated policies had far-reaching adverse collateral effects for various age groups. However, few large-scale studies have examined how the pandemic shaped children’s motor performance trajectories or how contextual factors, such as socioeconomic background (SEB) or recent secular trends, may have influenced these effects.

### Physical (In)activity in Children and Motor Development During Pandemic

Physical inactivity in children, already considered an epidemic [8], has been identified as one of the adverse consequences associated with the COVID-19 pandemic [9]. School and sports club closures during the nationwide lockdowns (LD I: March–May 2020; LD II: January–May 2021) removed many opportunities for organized physical activity, and restricted access to recreational spaces further reduced everyday physical activity [10, 11]. These reductions have been linked to changes in body composition and motor skill development [12], which is concerning given that lower physical activity in childhood is associated with lower activity and poorer health outcomes later in life [13]. Within the physical literacy framework, motor performance is understood as a multidimensional construct including endurance, strength, coordination, and flexibility, each of which follows domain-specific developmental trajectories [14–16]. Some domains (e.g., flexibility) tend to progress relatively continuously, whereas others (e.g., coordination or endurance) are more sensitive to environmental conditions and periods of developmental plasticity [13, 17]. Because the pandemic overlapped with such sensitive periods, domain-specific changes are plausible, with some motor abilities being more affected than others. Empirical findings to date are mixed [18], some studies report overall declines in children’s motor performance [19], with differences in magnitude and/or significance across motor domains [20, 21], while some find negligible overall change [22]. These inconsistencies highlight the need for large-scale data that consider domain-specific trajectories rather than relying on aggregate motor performance scores.

### Moderating Effects of Children’s SEB

Children’s socioeconomic background (SEB) is a key determinant of physical activity and motor development [23]. On average, children from lower SEB tend to show lower motor performance than peers from higher SEB [24], which is often attributed to differences in parental knowledge, available time, and financial resources for supporting active lifestyles [25]. During the pandemic, such disparities may have been reinforced if families with higher educational or material resources were better able to compensate for the loss of organized physical activity opportunities. At the same time, a narrowing of differences is also conceivable if children from higher SEB experienced greater reductions from previously higher activity levels. In addition, SEB-related housing and neighborhood conditions may have influenced access to safe indoor or outdoor spaces for movement during lockdowns [26]. Overall, the role of SEB in shaping pandemic-related changes in motor development remains unclear, underscoring the need to examine potential SEB-related moderation of motor performance trajectories.

### Considering Recent Secular Trends in Children’s Motor Development

When interpreting pandemic-related changes, it is essential to consider underlying secular trends, that is, long-term developments in children’s motor performance that may have preceded the pandemic. Pre-pandemic studies have reported heterogeneous trends in children’s physical fitness over recent decades, including gradual declines as well as periods of stability or improvement [17, 27–31]. In Germany, previous data from the ‘Berlin hat Talent’ (BHT) project further indicated sex- and SEB-related performance differences and suggested relatively stagnating or slightly declining motor fitness levels in recent years [32–35]. These findings provide an important local baseline for assessing the pandemic’s potential effects.

Recent analyses have begun to account for secular trends when assessing pandemic-related changes in children’s motor performance [20, 21], and broader reviews likewise highlight the heterogeneity of findings across settings and age groups [36]. However, these approaches typically relied on a single pre-pandemic baseline trend. Because estimated trends vary depending on the years selected, relying on one trend line may still over- or underestimate deviations during the pandemic. To address this, we modelled multiple pre-pandemic trend trajectories starting from different reference years to estimate expected motor performance in the absence of the pandemic. Comparing these expected trajectories with observed values allows a more robust assessment of how motor performance during and after the lockdown periods diverged from ongoing developmental trends. This approach provides a more nuanced and context-sensitive approximation of potential pandemic-related changes in children’s motor performance.

### Study Aim and Hypotheses

This study aimed to examine how the COVID-19 pandemic was associated with changes in children’s motor performance. We used data from the BHT project, a cohort-sequence study that has annually assessed third-grade children in Berlin since 2011, including the pandemic years 2020–2022. Specifically, we investigated whether motor performance after the two nationwide lockdowns differed from pre-pandemic years, whether any differences varied by motor domain, and whether these patterns were moderated by school-level socioeconomic background (SEB). We formulated three a priori hypotheses. First, we expected children’s motor performance to be lower after the lockdown periods compared to pre-pandemic years (H1). Second, we expected non-directional differences across motor domains, meaning that some domains may show stronger deviations than others, without specifying the direction or magnitude of these differences due to limited evidence to date (H2). Third, we expected that the association between lockdown periods and motor performance would be moderated by school-level SEB (H3), acknowledging that both widening and narrowing of disparities were theoretically plausible. To ensure that observed differences were not attributable to ongoing secular trends in motor performance, we additionally modelled multiple pre-pandemic trajectories and compared expected values with observed performance during the pandemic.

## Methods

### Study Characteristics and Participants

**Fig. 1:**
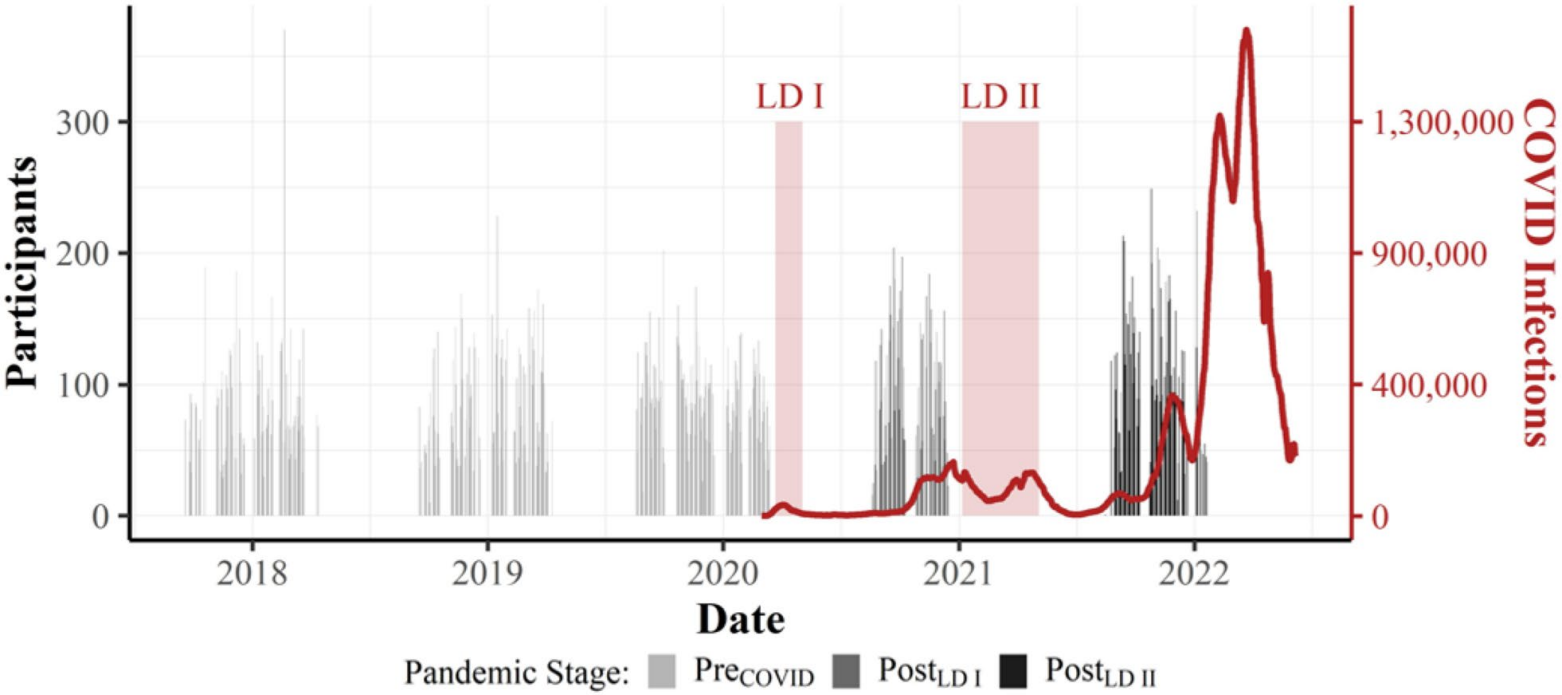
Overview of the number of third graders assessed in the 3 years before COVID-19 and after the two major Lockdown periods in Germany (LD I and LD II) from 2018 to 2022. The figure illustrates the dates of assessments throughout the year [12]. Assessments prior to 08/2018 are not shown here because the date of the assessment was not available. The two LD periods are highlighted as transparent red areas. Light gray color indicates assessments before COVID (Pre_Covid_, left), darker gray color indicates assessments after LD I (Post_LD I_), dark gray color indicates assessments after LD II (Post_LD II_). The prevalence of COVID-19, i.e., the number of actively infected individuals, is shown as red solid line

The current study was funded by the German Research Foundation (DFG) as a secondary analysis of data from the independent BHT program. BHT is an initiative of the Landessportbund Berlin (LSB) and the Berlin senate that aims to promote more specialized physical activity programs to facilitate physical fitness and activity in children with compromised or superior sports performance. The program started in 2011 and since then follows a quasi-longitudinal panel design (e.g., [32–35]). Each year, all public primary schools in Berlin are invited to participate, and third-grade students are tested for motor performance using the German Motor Fitness Test (GMT; [37, 38]). Participating schools represent diverse districts across Berlin, encompassing a wide range of socioeconomic backgrounds. Before participation, the schools received a written invitation from the Berlin school administration to participate in the project. The decision to participate in the program was at the discretion of the individual schools. Data assessment was then carried out by a professional assessment team (Maximum Sport GmbH, Berlin) with trained assessors over the past 10 years. All children eligible for the GMT were included, without additional inclusion or exclusion criteria. Participation was voluntary, with no compensation, and written informed consent was obtained from parents. The study was approved by the Senate of Berlin and as part of the DFG grant application. This study was not preregistered. Reporting followed STROBE guidelines [39]. In total, data from 68,996 third-grade children (mean age = 8.83 ± 0.56 years, range = 6.4–13.0; 35,270 girls, 51.1%) assessed between the 2011/2012 and 2021/2022 school years were analyzed (Table 1, Fig. 1). All participating children were enrolled in the third grade at the time of testing. The observed age range (6.4–13.0 years) reflects natural variation within this grade level, which is common in Berlin due to differences in school entry age and grade repetition. No motor performance data were missing. SEB information was unavailable for 777 children and was addressed as described in the Statistical Analyses section. Although sample sizes were not determined a priori, they are comparable to or larger than those in previous BHT reports.

**Table 1:** Characteristics of Study Participants by School Year

School Year	Participants (% female)	Age (Mean)	Age (SD)	Assessment period (if available)
2011/2012	2357 (47.98%)	8.54	0.64	Not available
2012/2013	2735 (50.16%)	8.60	0.68	Not available
2013/2014	3428 (48.22%)	8.36	0.61	Not available
2014/2015	5403 (47.78%)	8.37	0.62	Not available
2015/2016	7147 (49.27%)	8.32	0.60	Not available
2016/2017	6078 (48.98%)	8.35	0.60	Not available
2017/2018	7373 (49.50%)	8.38	0.64	Sep 2017–Apr 2018
2018/2019	8620 (49.03%)	8.42	0.65	Sep 2018–Apr 2019
2019/2020	10,482 (48.62%)	8.35	0.61	Aug 2019–Mar 2020
2020/2021	6607 (49.13%)	8.24	0.54	Aug 2020–Dec 2020
2021/2022	8766 (48.72%)	8.28	0.55	Aug 2021–Jan 2022

The number of participants recruited per school year is reported, along with the mean and standard deviation (SD) for participant’s age. Assessment dates are included for the years when this information was available

### Measures: German Motor Fitness Test & Socioeconomic Background

The German Motor Fitness Test (GMT; [37, 38]) is a standardized battery recommended by the German Association for Sport Science to assess motor performance and physical fitness in children and adolescents aged 6–18 years. It has been repeatedly validated for six test items—6-min run, sit-ups, push-ups, standing long jump, 20-m sprint, and side jumps—covering key domains of motor performance, including cardiorespiratory fitness, strength, speed, and coordination [16, 40].

*Cardiorespiratory fitness* was assessed using the 6-min run test. Participants had to run around a volleyball court (54 m) for 6 min. They motivated the children, who were instructed to complete as many rounds as possible. The distance covered within 6 min was measured for each subject. The covered distance (in m), derived from the number of laps completed within 6 min, plus the distance covered in the last lap was used for analysis.

*Strength* (*or Strength-Endurance*) was assessed by performing sit-ups, push-ups, and long-jump, which require muscular endurance of the trunk and lower and upper extremities musculature. For sit-ups, participants lay down on their back. They were asked to put down their feet on the floor, and to bend their knees. The experimenter held down the feet during testing and made sure that the knees were bent by approx. 80°. The hands had to be placed at the temples. Sit-ups were performed by raising the upper body from the supine position until both knees could be touched with the elbows, after which they had to return to the supine position with both shoulders touching the mat. Both feet had to be placed on the floor during sit-up performance. For push-ups, participants were instructed to start from a lying position with both hands fold together behind their back. After the start command, they had to do push-ups by placing their hands next to their shoulders, pushing off the ground, then releasing one hand from the ground to touch the other hand, and then returning to the starting position. Knees were not allowed to be placed on the floor during push-up performance. For both sit-ups and push-ups participants were instructed to complete as many repetitions as possible within 40 s. One trial was performed per test. The experimenter controlled for correct performance. Outcome measure was the number of correct repetitions (n) within 40 s. Next to these tests, jumping strength was measured using the standing long-jump test. Participants were asked to stand at a starting markup line with their feet parallelly as far as possible to the markup line. They then had to jump as wide as possible starting from that position, and they were instructed to land with both feet simultaneously. Two runs were performed. The outcome measure was defined as the distance (in cm) between their landing position at the heel of their rear foot and the markup line. The better of two trials was used for analysis.

*Speed* was measured with the 20-m sprint test. The participants started from a standing position. After a short starting command, the participants were instructed to run as fast as possible over a distance of 20 m. Two runs were performed. False starts were stopped and repeated by the experimenter. Time was measured with a hand-held stopwatch (accuracy 1/100 of a second). The experimenters started the time measurement synchronously with the starting command and stopped the time measurement when children crossed the finish line. Performance was defined as the time (in seconds) taken to run the 20 m. The better of two runs was used for analysis.

*Coordination* under time pressure was measured using the side jumping test. Participants were instructed to jump sideways across a mark-up line (about 5 cm width) centered to their body (sagittal) and that was placed on a rubber mat (size: 50 cm × 100 cm). They were asked to jump with both legs simultaneously and to try to cross the line as often as possible while not stepping on the markup line or crossing the marked area of the rubber mat. Two runs at 15 s each were performed, with a short rest period of at least 60 s to recover. The outcome measure was the number of jumps (n) completed within 15 s. The average of the two runs was analyzed.

*SEB* of children was derived at the school level, which was determined using the official but confidential school type classifications of the state of Berlin. Here, each school type is defined by various SEB characteristics of the school’s catchment area, such as parental income or employment rate. These aspects are summarized in a school type score ranging from 1 (very low SEB) to 7 (very high SEB). However, in the present sample, too few schools had a school type of 1 or 7. Therefore, these two types were combined with school types 2 and 6, respectively. The final school type score (1 very low SEB to 5 very high SEB) was used in the statistical analysis.

### Procedure

After schools agreed to participate in the BHT program, a testing date was scheduled for a professional assessment team to visit the school. Testing was conducted primarily between August and April to avoid overlap with spring and summer vacations (April–August) and minimize student absences. All testing equipment was provided by the assessment team and set up in a gymnasium or large multipurpose room prior to arrival. At each session, all children from a participating class received standardized instructions about the overall procedure and were then divided into eight groups. Each group began at a different testing station and rotated sequentially through all eight stations until all tests were completed. Examiners remained at their assigned stations throughout and recorded each child’s performance, which was subsequently entered into a predefined central database by the assessment team. The same standardized testing procedures were applied before and during the pandemic, including the post-lockdown assessment periods following LD I (March–May 2020) and LD II (January–May 2022; see Fig. 1).

### Data Analysis and Statistics

All data analyses and statistics were performed with R Studio (v 4.2.1; [41]).

First, raw data on motor performance were categorized into percentile values based on German norm values from the MoMo study ([42]; 6-min run norms were not published, but were provided for our study) per test measure (6 min run, sit-ups, push-ups, long-jump, 20 m run, jumping sideways) and biological sex (female, male). This transformation is crucial when examining secular trends across years, as underlying performance trends can affect the interpretation of other influences, such as pandemic effects. Percentile values were standardized to z-scores for analysis and back-transformed to quantile-normalized percentiles for interpretation. Analyses were designed to examine pandemic-related differences in children’s motor performance, with particular focus on time-related changes, moderated by motor domains, and SEB. Analyses were conducted in two stages: (1) comparing pre- and post-pandemic performance, and (2) a sensitivity analysis adjusting for secular trends by comparing actual and predicted performance trajectories. Additional sensitivity analyses examined the effects of body size and outliers, (Additional file 1: Tables S4–S6). Also, quadratic effects of Time were tested to evaluate potential non-linearity (Additional file 1: Table S7); as these did not improve model fit, a linear specification was retained. The results of these analyses indicate that the outcomes of our models remained robust despite variations in the input data.

#### Comparison of Motor Performance Before and During the Pandemic

In the first analysis approach, we performed a cross-classified linear mixed effects model (‘lme4’ package; [43]) with all data available (2011/12 to 2022/23). Test scores (level 1) were nested in motor domains (level 2a) and in participants (level 2b) and participants were nested in schools (level 3b). Here, we analyzed the difference between pre-pandemic motor performance and motor performance after LD I and LD II (H1). Pre-pandemic motor performance was calculated as average motor performance across all pre-pandemic years (August 2011/12–March 2019/20). Secular trends were not yet considered at this point. The dependent variable was motor performance (z-values, continuous scale). Further, level 2 and level 3 variables were included in the model: Sex (level 2; male, female), Time (level 2; pre, post LD I, post LD II), and SEB (level 3; continuous integers from 1 to 5, higher values indicate higher SEB) as well as all interactions between independent variables, i.e., two-way and three-way, were added to the model as fixed effects. Further covariates were not added. For the random effects term, random intercepts were added for Motor Domain and School, with Participants nested in Schools. By including random intercepts, the model acknowledges that measurements taken from the same participant or same school are more likely to be similar (correlated) than measurements from different participants or schools. This is crucial for within subject factors (here Motor Domain) data to accurately estimate the effect of interest while accounting for the structure of the data. The model was fitted using maximum likelihood estimation (ML), which is assumed to provide better estimates for fixed effects than restricted maximum likelihood estimation (REML). Crucially, ML is capable of providing unbiased estimates despite the presence of missing data, by leveraging all available information and operating under the assumption that data are missing at random (MAR). This approach ensures a more efficient and comprehensive use of the dataset [44]. In addition to the model estimated, F-statistics were obtained for main and interaction effects using type-III sum of squares ANOVA (“anova”; R base package) with Satterthwaite-Approximation, a slightly more liberal procedure than the Kenward–Roger method [45]. All tests were performed two-sided and significance level was set to α < 0.05. Post-hoc tests were performed on significant main and interaction effects using planned contrasts with estimated marginal means (for factor variables) and estimated marginal trends (for continuous variables) both from the *emmeans* package [46]. Only effects involving Time were followed up by planned contrasts; other interactions (e.g., Motor Domain × SEB) were not examined further. Post-hoc tests were designed to support the main and interaction effects and provide a more detailed picture of the differences between the individual levels of the multi-level factor variables of interest.

#### Trend Analysis: Differences Between Actual and Predicted Motor Performance During the Pandemic

The second part of our statistical analysis aimed at considering secular trends in investigating the impact of the pandemic on motor performance. Here, we aimed to use the secular trends to make the most accurate predictions of what motor performance the children would have achieved without the influence of the pandemic. These predictions can then be compared to the children’s actual motor performance during the pandemic to better assess the true impact of the pandemic. However, different secular trends may result in varying predictions depending on the data/years used. To also account for this variability in secular and varying predictions, respectively, different starting years (2011–2017) were used in an iterative process to predict motor performance during the pandemic school years.

We first estimated linear secular trends (Year, continuous; 2011/12–2019/20) per Motor Domain and SEB (5-level factor) using cross-classified LMMs (z-scaled outcome; ML) with Sex as a covariate and random intercepts for School. Starting years were iteratively shifted (2011–2018) to capture variability in trend estimation. We then predicted performance for 2020/21 and 2021/22 (post-LD I/LD II) and, for each child, compared predicted vs. actual values in additional LMMs with Type (predicted, actual) as a fixed effect and random intercepts for Participant. In total, 480 models (2 LDs × 6 motor domains × 5 SEB × 8 starting years) yielded difference estimates that were analyzed in a final LMM testing LD, Motor Domain, and SEB effects; these cell-wise models were used to obtain estimates rather than to draw direct statistical inferences.

Finally, all these 480 model estimates were forwarded to the final analysis investigating the true effect of the pandemic on motor performance. That is, a final linear mixed effects model was performed. The outcome measure was the estimate of the difference between predicted and actual motor performance (N = 480). In the first step, we only added random intercepts for Starting Year of the secular trend. Here, we examined the significance of the intercept, to evaluate whether the estimates of the difference between predicted and actual motor performance are not equal to zero; thus indicating a non-zero overall effect of the pandemic related LD on motor performance (H1). After that, we also added fixed effects for the independent variables, LD (LD I and LD II), Motor Domain (6-level factor), and SEB (5-level factor), and their interactions, to investigate whether pandemic-related differences in motor performance would vary between Motor Domains (H2), and differential school-level SEB (H3). ML was applied for estimation of fixed effects. 

All statistical tests were conducted as one-sided, based on the a priori specification of directional hypotheses. Only significant main and interaction effects including Time (i.e., *p* < 0.05) were followed up with post-hoc tests, using planned contrast with estimated marginal means. The reason was that we were only interested in pandemic effects (i.e. Time-related) in this study, while not in general differences in motor performance between Motor Domains or SEB. Partial eta-squared (*η*^*2*^) is reported as a measure of effect size for main and interaction effects, using the *effectsize* package in R (Ben-Shachar et al., 2020). It provides an interpretable index of the proportion of variance associated with each fixed effect, facilitating comparisons with previous studies. In the literature, eta-squared (η^2^) is commonly interpreted as indicating small (< 0.06), moderate (0.06–0.14), or large (> 0.14) effect sizes. However, we caution against strict adherence to these conventional thresholds, as they may not accurately capture the actual impact of the effects under investigation. Consequently, we emphasize the importance of interpreting effect sizes within the broader context of the study and its practical significance [47]. Z-scores from mixed models were back-transformed to quantile-normalized percentiles for further practical interpretation; confidence intervals for marginal means are provided in Additional file 1: Table S3.

**Fig. 2:**
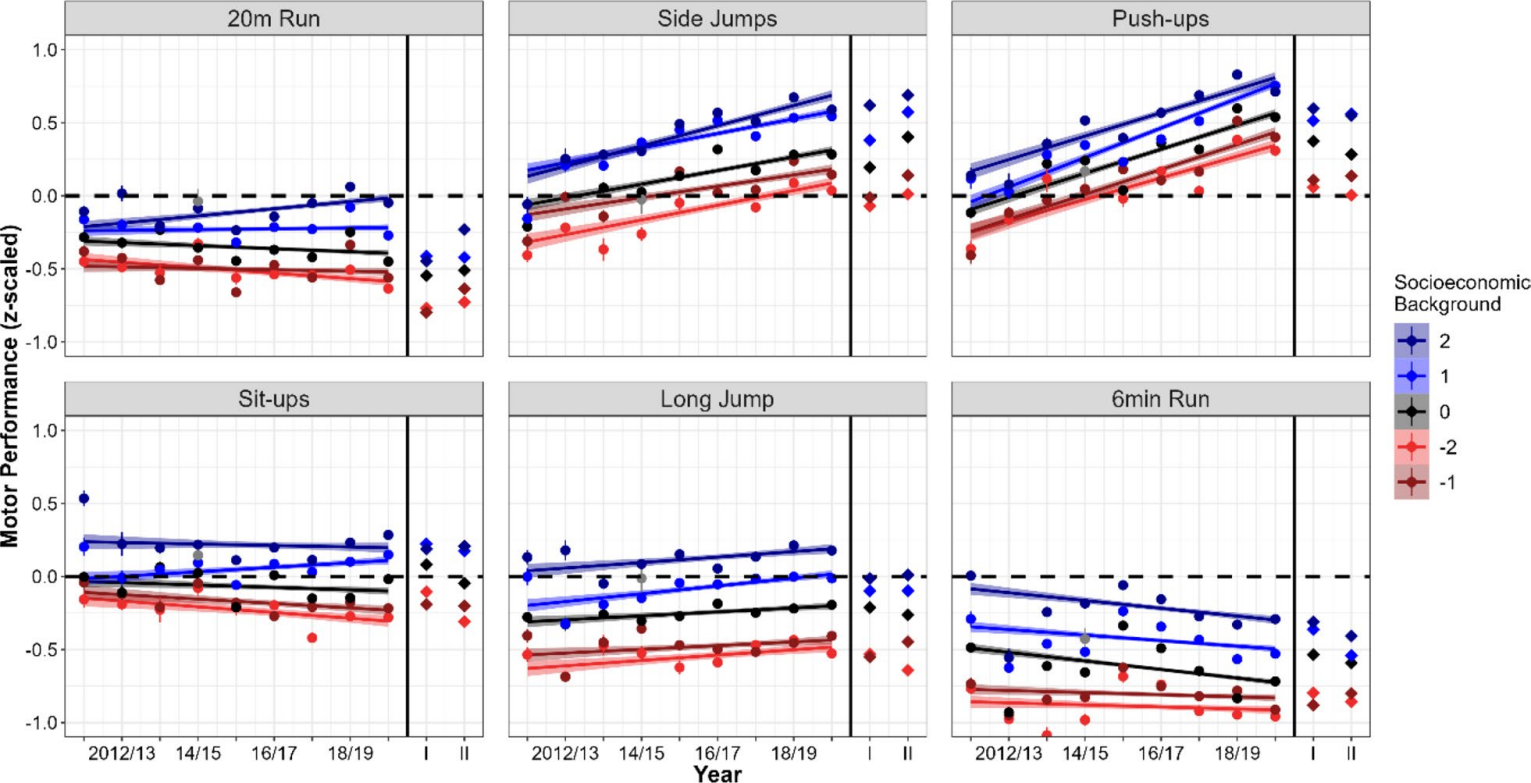
Secular trends in children’s motor performance from 2011 to 2019 (n = 53,570) and during the pandemic lockdowns (LD I and LD II; n = 15,426), displayed separately by motor domain (panel) and school-level socioeconomic background (SEB; color). The y-axis represents average motor performance (z-transformed, quantile-normalized percentile values), and the x-axis represents school years from 2011/12 to 2022/23. Linear secular trends (solid lines) were estimated from pre-pandemic data (2011/12–2019/20; circular points) for each domain and SEB group. Motor performance values during the pandemic (rhombs) are plotted to the right of the vertical black line. Error margins represent standard errors. The figure illustrates that motor performance declined during and after the pandemic lockdowns across most domains and SEB levels, deviating from the pre-pandemic trend projections

Please note that we controlled only for the effects of Sex in our analysis, because boys and girls can exhibit different rates of physical development and varying levels of motor performance due to biological and social factors, but we were not interested in the effects of Sex as part of our main research questions. For interested readers, these additional analyses on the effects of Sex and its interactions with our main variables of interest (Time, Motor Domain, SEB) can be found in the supplementary materials of this article, specifically Additional file 1: Table S1 and Figs. 1 and 2. Importantly, no significant interactions involving Sex and Time were observed, suggesting that the pandemic's impact was consistent across both sexes. This observation lends further credence to the robustness of our findings.

## Results

### Motor Performance During Covid-19 Compared to Pre-Pandemic

A cross-classified linear mixed effects model was performed to investigate all three hypotheses together, i.e., the effects of Time (H1), Time × Motor Domain (H2), Time x Motor Domain × SEB (H3).

First, we observed a significant main effect of Time (H1), *F*(2, 32,199) = 3.59, *p* = 0.014, η^2^ < 0.01. Post-hoc contrasts revealed lower motor performance at LD II compared to pre (*p* = 0.031). Differences between LD II and LD I and between LD I and pre were not significant.

The main effect of the Motor Domain, *F*(5, 335,151) = 7,102.35, *p* < 0.001, η^2^ = 0.10, was significant, indicating different motor performance levels across different Motor Domains. The main effect of SEB was also significant, *F*(1, 789) = 450.50, *p* < 0.001, η^2^ = 0.36, indicating that children from lower SEB generally had worse levels of motor performance than children from higher SEB, respectively.

In addition to these main effects, we also found significant interactions, including an interaction of Time × Motor Domain (H2), *F*(10, 335,085) = 117.85, *p* = 0.001, η^2^ < 0.01. Pairwise post-hoc contrasts revealed differential effects of Time between Motor Domains (see Additional file 1:Table S2): For the 20 m run, performance was lower at LD I (*p* < 0.001) and LD II (*p* < 0.001) compared to pre, and lower at LD II compared to LD I (*p* = 0.037). For side jumps, performance was lower at pre and LD I compared to LD II (*p* < 0.001), and there was no difference between pre and LD I. For push-ups, there was no significant difference between pre, LD I, and LD II. For sit-ups, in contrast, performance was lower at pre compared to LD I (*p* < 0.005) and performance at LD I was higher compared to LD II (*p* < 0.001), with no significant difference between pre and LD II. For the long-jump, performance was higher at pre compared to LD I (*p* = 0.023) and LD II (*p* = 0.001), but there was no difference between LD I and LD II. Lastly, the performance of the 6 min run was lower at pre compared to LD I (*p* = 0.016) but higher compared to LD II (*p* = 0.001), and higher at LD I compared to LD II (*p* < 0.001). While the interaction of Motor Domain × SEB was significant, *F*(5, 335,055) = 26.92, *p* < 0.001, η^2^ < 0.01., the interaction of Time × SEB was not significant, *F*(2, 36,451) = 0.48, *p* = 0.309, η^2^ = 0.01, indicating the overall effect of SEB on motor performance was similar at pre, LD I and LD II. However, the triple interaction of Time × Motor Domain × SEB was significant (H3), *F*(10, 335,059) = 9.67, *p* < 0.001, η^2^ = 0.01. Post-hoc trend analysis (see Additional file 1:Table S3) revealed larger differences, i.e., more differential trends, in motor performance between children from lower compared to higher SEB. For the 20 m run, the effect of SEB was not significantly different between pre, LD I and LD II. For side jumps, the effect of SEB was stronger at LD II compared to pre (*p* = 0.005), but both LD II and pre were not different from LD I. Likewise, for push-ups, the effect of SEB was more pronounced at LD II compared to pre (*p* = 0.009), but there was no difference between pre and LD I or between LD I and LD II. For sit-ups, the effect of SEB on motor performance was also higher at LD II compared to pre (*p* = 0.044) and compared to LD I (*p* = 0.022). However, there was no difference between pre and LD I. For the long-jump, there was an inverse effect, such that the trend of SEB was higher at pre compared to LD I (*p* = 0.015), but there was no difference in trends between pre and LD II or between LD I and LD II. Lastly, for 6 min run, there was a higher effect of SEB on motor performance at pre compared to LD II (*p* < 0.001), but neither a significant difference between pre and LD I nor between LD I and LD II.

### Predicted vs Actual Motor Performance Based on Different Secular Trends

Figure 2 illustrates the rationale for considering secular trends. Data shows secular trends in motor performance from 2011 to 2019 (linear predictions) per Motor Domain (boxes) and SEB (colors), and actual motor performance during the pandemic (rhombs). As Fig. 2 shows, motor performance during the pandemic should not be compared only to average motor performance in previous years (e.g., average between 2017–2019 or 2011–2019), because analyses do not account for recent secular trends in specific Motor Domains. These data suggest that recent trends in motor performance would have resulted in higher (e.g., push-ups) or lower (e.g., 6-min run) motor performance in the two years of the pandemic. Therefore, not including these trends (as in the analyses above) in statistical analyses could lead to a biased estimation (underestimation or overestimation) of the impact of the pandemic on motor performance.

In the second part of our analysis, predicted motor performance values from varying secular trends were compared to actual motor performance values during pandemic years.

First, we found that the intercept was significantly different from zero, *t*(7) = − 5.39, *p* < 0.001, β = − 0.10, − 3.85%. That is, the actual motor performance during pandemic (across all motor domains and SEB) was significantly different from the motor performance predicted from secular trends. The main effect of LD, however, was not significant, *F*(1, 413) = 2.09, *p* = 0.074, η^2^ < 0.01. That is, there was no significant difference between LD I and LD II in their overall effect on motor performance. However, the main effect of Motor Domain was significant, *F*(5, 413) = 182.06, *p* < 0.001, η^2^ = 0.69, indicating differential effects of the pandemic on individual Motor Domains (for further details cf. Additional file 1: Table S2): Push-ups were affected most negatively (β = − 0.40, − 15.47%), followed by 20 m run (β = − 0.26, − 10.32%), long-jumps (β = − 0.12, − 4.63%), and side-jumps (β = − 0.06, − 2.58%). The two other domains, 6 min run (β = 0.19, 7.56%) and sit-ups (β = 0.07, 2.75%) increased during pandemic. The effect of SEB also was significant, *F*(4, 413) = 57.48, *p* < 0.001, η^2^ = 0.36. The greater negative effect was found for children from very high SEB (β = − 0.22, − 8.56%), followed by children from high SEB (β = − 0.15, − 5.85%), children from low SEB (β = − 0.15, − 5.80%), and children from average SEB (β = − 0.04, − 1.50%). Children from very low SEB descriptively showed higher actual motor performance than predicted motor performance (β = 0.06, 2.51%). Post-hoc contrasts between the five levels of SEB can be found in the supplement (Additional file 1: Table S3).

**Fig. 3:**
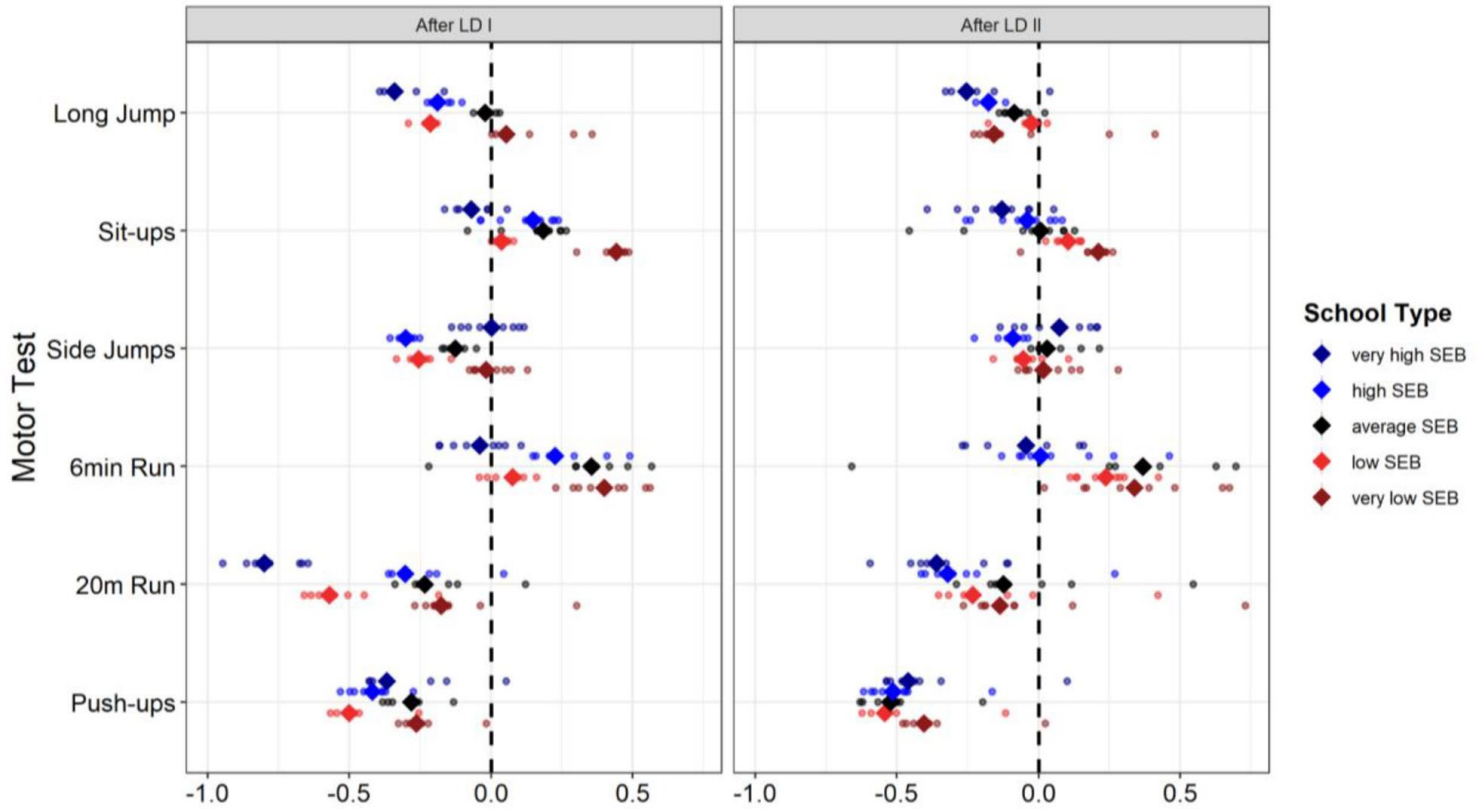
Predicted (dotted line) and observed (colored dots) motor performance during the two pandemic lockdowns—LD I (n = 6,660; left) and LD II (n = 8,766; right)—across motor domains (y-axis) and school-level socioeconomic background (SEB; color). Predicted values are based on multiple pre-pandemic secular trend models using different starting years. Rhombs reflect the median prediction across models. Data points positioned to the left of the black prediction line indicate lower observed than predicted motor performance; points to the right indicate higher observed performance. The figure demonstrates that observed performance levels during both lockdowns were consistently below the predicted values across most domains, confirming the robustness of the trend-adjusted pandemic effects

In addition to these main effects, all interactions were significant, including the interaction of LD x Motor Domain, *F*(5, 413) = 18.94, *p* < 001, η^2^ = 0.19. Post-hoc revealed that LD I had a stronger effect than LD II on push-ups (*p* < 0.001) and sit-ups (*p* < 0.001), and that LD II had a more pronounced effect than LD I on 20 m run (*p* < 0.001) and side jumps (*p* < 0.001). There was no difference between LD I and LD II in their effects on 6 min run (*p* = 0.271) and long-jump (*p* = 0.333), respectively. Furthermore, the interaction of LD × SEB was significant, *F*(4, 413) = 11.24, *p* < 0.001, η^2^ = 0.10. Post-hoc tests showed that during LD I had a stronger effect than LD II on motor performance in children with very low SEB (*p* = 0.007), and that LD II had a more pronounced effect on LD I on children with low SEB (*p* < 0.001) and very high SEB (*p* = 0.008). There was no difference between the effects of LD I and LD II on motor performance in children from average SEB and high SEB. Moreover, the interaction of Motor Domain x SEB, *F*(20, 413) = 7.28, *p* < 0.001, η^2^ = 0.26, was significant. Lastly, also the triple interaction of LD x Motor Domain x SEB was significant, *F*(20, 413) = 1.68, *p* = 0.017, η^2^ = 0.08, indicating that the two LD had differential effects on specific Motor Domains that further varied between different SEBs (Fig. 3).

## Discussion

The aim of this study was to examine whether motor performance in third-grade children differed between pre- and post-pandemic cohorts, while also considering SEB and secular trends in motor development. In line with H1, analyses that compared cohort means without considering secular trends suggested small and mixed differences between pre- and post-pandemic cohorts across motor domains. However, several motor domains in this population showed positive developmental trajectories prior to the pandemic, meaning that children assessed in later years would typically be expected to perform better, not similarly or worse. When secular trends were incorporated to estimate the counterfactual developmental trajectory, that is, the level of performance expected had typical progression continued, the magnitude became larger and pattern of differences changed substantially. Consistent with H2, interruptions in expected motor development varied. Negative effects of the pandemic were particularly evident for domains requiring coordinated and speeded movement: upper-body strength (push-ups) was − 15.47% lower and speed (20 m sprint) was – 10.32% lower than anticipated. In contrast, endurance (6-min run: + 7.56%) and core strength (sit-ups: + 2.75%) showed small increases, which likely reflect changes in daily physical activity behaviour during lockdown. Lower-limb power (standing long jump) and coordination (side jumps) showed comparatively smaller deviations from expected developmental progressions. In line with H3, these trend-adjusted shortfalls varied between children from different SEB, but they showed to be larger among children from higher SEB backgrounds overall (e.g., very high SEB ≈ − 8.56%, high SEB ≈ − 5.85%), while very low SEB showed a small positive deviation (+ 2.51%), although moderation patterns varied by motor domain and lockdown stage. In summary, the pandemic seemed to have various effects on motor performance, some of which were strongly negative (e.g., push-up or 20 m run performance), while others were even slightly positive (e.g., 6 min run or smaller SEB gap). Further steps are now to consider, develop, and implement specialized (physical activity and motor performance) programs to rehabilitate the performance losses in the most compromised motor domains and provide solutions to facilitate motor performance across children from different SEB. To guide interpretation, the following discussion focusses on the trend-adjusted estimates, as these reflect expected developmental progression and provide a more meaningful reference point than raw cohort averages.

### General Discussion

Consistent with previous research, we found an overall lower motor performance in children during the pandemic compared to motor performance in children before the pandemic [48, 49]. Although the domain-general difference between pre-pandemic and post-pandemic motor performance, on average, appeared to be rather small (-4%), it should be kept in mind that even such small overall differences can have serious and persistent negative consequences for various health-related factors, for childhood, adolescence, and beyond. For example, lower levels of physical fitness and activity have been found to lead to lower motivation for physical activity in later adulthood, which in turn may affect physical, cognitive, and psychosocial health [50–52]. Further, it should be noted that from a developmental perspective, 4% equals about one year of development (cf. [42]). Estimating the delay in motor development that has occurred in response to the pandemic is important in itself, but also because delayed motor development may compromise development in other domains, such as cognitive, social, and emotional development [53–55].

Interpretation of these results should also take into account that the pandemic seemed to have varying effects on different motor domains, both positive and negative [49]. One plausible explanation is that domains such as upper-body strength (push-ups) and sprint speed (20 m run), which typically benefit from structured practice and instructional feedback in physical education and organized sport, may have been particularly vulnerable when these settings were unavailable during pandemic. In third-graders, this period coincides with some consolidating coordination and strength capacities, suggesting that the interruption may have occurred during developmentally sensitive phases, despite this issue has been discussed critically in recent years [56–58]. Although our design does not permit causal inference, the pattern we observed, i.e., larger shortfalls in these domains relative to expected developmental progression, is consistent with this interpretation, and with previous research [21]. In contrast, endurance (6-min run) and core strength (sit-ups) showed small increases, which may reflect greater opportunities for unstructured outdoor or family-based physical activity during lockdowns. These activities support aerobic endurance more readily than coordination- or speed-intensive skills, which may help explain why these domains showed relative improvements. Meanwhile, coordination (side jumps) and lower-limb power (standing long jump) appeared comparatively stable, which could indicate that they were partly supported by maturational processes and incidental physical activity [57, 58]. However, further research is needed to confirm this assumption. The long-term implications of these domain-specific patterns remain uncertain. If the observed differences primarily reflect delayed progression rather than permanent loss, opportunities to re-engage children in structured physical activity through physical education and community sport may help restore typical developmental trajectories. But we need continued monitoring and targeted support programs, including school-based initiatives [59] and technology-supported activity formats [60], for assessing whether these developmental differences diminish, stabilize, or widen over time.

Another important finding is the moderating effect of children’s SEB on pandemic-related changes in motor domains. While there was no overall effect of children’s SEB on pandemic-related differences in motor performance, individual motor domains were differentially affected. For side jumps, push-ups, and sit-ups, the performance gap between children with lower and higher SEB increased, whereas for long-jump and the 6-min run, the gap decreased. This pattern of results could also be related to a change in the children’s physical activity behavior, including their SEB. Children with higher SEB were more likely to be active in sports clubs, whereas children with lower SEB were less likely to be physically active in sports clubs. Because sports clubs were largely closed during the pandemic, children with higher SEB could not maintain their physical activity levels, while children with lower SEB remained similarly (in)active. This means that children with a higher SEB showed greater deficits in their motor development because they started from a higher baseline level, so that the gap closed to a certain extent. However, it is very likely that other SEB-related factors had differential effects on physical activity and Motor Domain changes during the pandemic, such as parental employment and income [61]. Children with higher SEB are more likely to have both parents employed, whereas children with lower SEB are more likely to have one parent unemployed who could have cared for the children during the pandemic and beyond, such as going to the playground [62]. Children with higher SEB, on the other hand, may be more likely to have stayed at home with their siblings or grandparents, resulting in lower levels of physical activity. However, it also should be considered that SEB was assessed at the school level. Potentially, the quality and focus of physical education classes may differ to some degree between schools with higher and lower school types, e.g., due to infrastructure. That is, physical education classes might be more likely to be more advanced and effective in schools with higher SEB, i.e. with catchment areas of higher SEB. The loss of higher-quality physical education could therefore have had a greater impact than the lack of lower-quality physical education, which also narrowed the gap between lower- and higher SEB. Future research should explore whether schools in higher SEB areas are more successful in recruiting specialized PE teachers compared to those in lower SEB regions, to understand the impact of socioeconomic factors on the distribution of PE staff.

In summary, this study offers two key contributions to the existing literature. First, by accounting for positive secular developmental trends, we were able to estimate expected motor performance and evaluate the pandemic period relative to the developmental trajectory rather than raw historical averages. This counterfactual approach helps distinguish temporary variation from disrupted growth, which has not been addressed in previous reports. Second, the finding that trend-adjusted interruptions were more pronounced among children from higher SEB backgrounds suggests a convergence of SEB disparities, indicating that pre-pandemic advantages in structured activity access and organized sport may have been temporarily reduced. These contributions extend current evidence by demonstrating that the developmental impact of the pandemic cannot be understood without situating performance within expected trajectories and considering how social gradients may shift under changing environmental conditions. The results of this study have several implications. First, given that impaired motor development can compromise various aspects of lifelong health, communities and policymakers should take effective action to compensate for the pandemic-based negative trends in children’s motor performance. Second, some SEB-related disparities in motor performance have decreased during the pandemic, although they may increase again as sport clubs and physical activity facilities have reopened. Consequently, authorities are called into action to promote and subsidize access to physical activity facilities and sport clubs, particularly for low SEB neighborhoods. Children from low SEB areas can thus be supported in bridging the gap to their peers from higher SEB neighborhoods. Third, further and comprehensive monitoring of children’s motor development in whole Germany is strongly needed in the future to a) evaluate the long-term impact of the pandemic in prospective years, and b) design and evaluate effective physical activity programs for children with compromised motor performance. This monitoring should pay special attention to specific motor domains within physical fitness and motor competence. Further, support programs are needed especially in neighborhoods with low SEB for whom decrements in motor performance levels were observed the most providing programs that are effective, tailored and individualized to the specific needs of the children.

### Strength & Limitations

The strength of this study is the large sample and the fact that motor performance was tracked since 2011 which allowed us to account for secular trends. Advanced statistical analyses were utilized to examine potential effects of COVID-19 related LD on children’s motor performance. The German Motor Fitness Test was used in this study, which is supported by both the German Government and the German Sport Association. However, this study is not without limitations. The data stems only from one large city and metropolitan area in Germany and is not representative for rural areas across Germany and other cultures/countries. Therefore, generalization should be limited to similar urban school environments with comparable activity infrastructures. The secular effects might result from political strategies which came together with the BHT project as local sports support groups were created in the different city districts and schools. Thus, while secular trend adjustment strengthens developmental interpretation, the specific drivers of these trends cannot be isolated. Socio-economic status was not measured on individual level, but at the school level, which may not reflect the heterogeneity of children within a school. This is particularly relevant for interpreting the SEB convergence finding, which should therefore be viewed as school-context–level rather than individual-level convergence. Another important limitation is the cohort-sequence design of this study, which does not include repeated measurement of the same children over the duration of the pandemic. Therefore, only the effects of the pandemic on different cohorts of children could be examined, but not the effects of the pandemic on cohort-specific developmental trends. As a result, developmental inferences reflect population-level trajectories rather than within-child change. Moreover, because the COVID-19 pandemic affected all children, a true experimental control group unaffected by the pandemic was not feasible. Instead, pre-pandemic cohorts served as a quasi-control condition. However, with our secular trend analysis, we approximated (various) expected motor performance trajectories in the absence of the pandemic, which showed a prominent difference between the actual and expected motor performance. Although results are very likely linked to the pandemic, it is not possible to make completely reliable statements about the causality of these effects.

### Conclusion

In conclusion, when motor performance was evaluated relative to expected developmental progression (recent secular trends), pandemic-related reductions (and some increments) became clear in specific domains. Trend-adjusted estimates showed that expected gains in upper-body strength and speed were interrupted, corresponding to meaningful delays in age-typical motor development. These effects were domain-specific, whereas endurance and core strength showed small increases likely reflecting compensatory shifts in everyday (physical) activity during periods of restricted sport participation. Trend-adjusted patterns also indicated a partial convergence across SEB, suggesting that pre-pandemic advantages linked to structured sport and organized activity in higher level SEB children may have been temporarily reduced. Methodologically, this study demonstrates the importance of incorporating secular developmental trends when evaluating population-level change, as raw cohort comparisons alone can underestimate or mischaracterize pandemic-related effects. Together, the findings indicate that COVID-19 did not produce a uniform decline in motor performance, but rather a disruption of expected developmental gains in specific skill domains, particularly those that depend on varied, practice-rich movement environments. Continued monitoring is needed to determine whether these developmental delays resolve, stabilize, or widen as children progress through later school years.

## Supplementary Information

Additional file 1.

## Data Availability

The data that support the findings of this study are available upon reasonable request from the principal investigators of the ‘Berlin hat Talent’ Study. Please contact the corresponding author for further information. The analysis code used to statistically test the data is under peer review and will be made available in a public repository after publication.

## Abbreviations

BHT: Berlin hat talent (longitudinal cohort study)
COVID-19: Coronavirus disease 2019
DFG: Deutsche forschungsgemeinschaft (German Research Foundation)
DMT: Deutscher motorik-test (German Motor Fitness Test)
GMT: German motor fitness test
LD: Lockdown
LD I: First lockdown (March–May 2020)
LD II: Second lockdown (January–May 2021)
MoMo: Motorik-modul study
PE: Physical education
SEB: Socioeconomic background
STROBE: Strengthening the reporting of observational studies in epidemiology
