# Supplementary material for: Motor Performance Before, During and After COVID-19 and the Role of Socioeconomic Background: A 10-Year Cohort Study of 68,996 Third Grade Children
Source: Sports Med Open. 2026 Jan 7;12:3. doi: 10.1186/s40798-025-00968-w (PMC12779792; doi:10.1186/s40798-025-00968-w)
Supplement: Supplementary file 1 — Additional file 1. [file 40798_2025_968_MOESM1_ESM.docx]

**Sports Medicine – Open**

Supplementary Material to:

Motor performance before, during and after COVID-19 and
the role of socioeconomic background: A 10-year cohort study
of 68,996 third grade children

Robert Stojan^1, 2*^, Katharina Utesch^3^, Ludwig Piesch^4^, Malte Jetzke^1^, Jochen Zinner^5^, Dirk Büsch^6^ & Till Utesch^4^

^1^ University of Münster, Muenster, Germany

^2^ Martin-Luther-University Halle Wittenberg, Halle, Germany

^3^ German Aerospace Center, Hamburg, Germany

^4^ [University of Luebeck](https://scholar.google.de/citations?view_op=view_org&hl=de&org=18300428284803003135), Luebeck, Germany

^5^ German University of Health and Sports, Berlin, Germany

^6^ University of Oldenburg, Oldenburg, Germany

**Corresponding author:*
Robert Stojan

[robert.stojan@sport.uni-halle.de](mailto:till.utesch@uni-luebeck.de)

**Supplementary material**

**Table 1** Additional exploratory analysis on the main and interaction effect of sex using linear mixed effects models

| *Term* | *F* | *NumDF* | *DenDF* | *p* | *pn²* |
| --- | --- | --- | --- | --- | --- |
| Time | 3.55 | 2 | 32276.38 | 0.03 | < 0.01 |
| SEB | 453.56 | 1 | 797.64 | 0.00 | 0.36 |
| Motor Domain | 7163.08 | 5 | 335140.67 | 0.00 | 0.10 |
| Sex | 361.01 | 1 | 68063.68 | 0.00 | 0.01 |
| Time x SEB | 0.47 | 2 | 36520.64 | 0.62 | < 0.01 |
| Time x Motor Domain | 118.92 | 10 | 335074.83 | 0.00 | < 0.01 |
| SEB x Motor Domain | 25.16 | 5 | 335047.64 | 0.00 | < 0.01 |
| Time x Sex | 1.58 | 2 | 68097.02 | 0.21 | < 0.01 |
| SEB x Sex | 37.09 | 1 | 67955.27 | 0.00 | < 0.01 |
| Motor Domain x Sex | 271.95 | 5 | 335147.77 | 0.00 | < 0.01 |
| Time x SEB x Motor Domain | 9.80 | 10 | 335051.70 | 0.00 | < 0.01 |
| Time x SEB x Sex | 0.17 | 2 | 68069.58 | 0.85 | < 0.01 |
| Time x Motor Domain x Sex | 1.81 | 10 | 335081.45 | 0.05 | < 0.01 |
| SEB x Motor Domain x Sex | 4.62 | 5 | 335054.60 | 0.00 | < 0.01 |
| Time x SEB x Motor Domain x Sex | 0.51 | 10 | 335058.57 | 0.89 | < 0.01 |

The supplementary analysis incorporates the interaction effect of Sex with the three independent variables: Time, Motor Domain, and Socioeconomic Background (SEB). These interactions were not included in our original research questions. However, considering their potential relevance to certain readers, we have included these results in the supplementary material

**Table 2** Pairwise Contrasts between Motor Domains on Motor Performance Differences Using Estimated Marginal Means

| Contrast | Estimate | SE | df | *t* | *p* |
| --- | --- | --- | --- | --- | --- |
| Pushups vs. 20m sprint | -0.14 | 0.02 | 413.00 | -6.08 | < 0.001 |
| Pushups vs. 6min Run | -0.59 | 0.02 | 413.00 | -26.22 | < 0.001 |
| Pushups vs. Jumping Sideways | -0.33 | 0.02 | 413.00 | -14.84 | < 0.001 |
| Pushups vs. Situps | -0.47 | 0.02 | 413.00 | -20.80 | < 0.001 |
| Pushups vs. Standing Broadjump | -0.28 | 0.02 | 413.00 | -12.55 | < 0.001 |
| 20m Sprint vs. 6min Run | -0.45 | 0.02 | 413.00 | -20.14 | < 0.001 |
| 20m Sprint vs. Jumping Sideways | -0.20 | 0.02 | 413.00 | -8.77 | < 0.001 |
| 20m Sprint vs. Situps | -0.33 | 0.02 | 413.00 | -14.73 | < 0.001 |
| 20m Sprint vs. Standing Broadjump | -0.15 | 0.02 | 413.00 | -6.47 | < 0.001 |
| 6min Run vs. Jumping Sideways | 0.26 | 0.02 | 413.00 | 11.38 | < 0.001 |
| 6min Run vs. Situps | 0.12 | 0.02 | 413.00 | 5.42 | < 0.001 |
| 6min Run vs. Standing Broadjump | 0.31 | 0.02 | 413.00 | 13.67 | < 0.001 |
| Jumping Sideways vs. Situps | -0.13 | 0.02 | 413.00 | -5.96 | < 0.001 |
| Jumping Sideways vs. Standing Broadjumpg | 0.05 | 0.02 | 413.00 | 2.29 | 0.200 |
| Situps vs. Standing Broadjumpg | 0.19 | 0.02 | 413.00 | 8.25 | < 0.001 |

Estimates are non-standardized

| **Table 3** Estimated differences between actual and predicted motor performance per motor domain and SEB after pandemic lockdowns (LD I and LD II) | | | | | | | |
| --- | --- | --- | --- | --- | --- | --- | --- |
| Lockdown | Motor Domain | SEB | emmean | Actual vs Predicted Motor Performance (in %) | lower.CL | upper.CL |  |
| After LD I | Pushups | very low | -0,241 | -9,51 | 0.14 | 0.34 |  |
| After LD II | Pushups | very low | -0,361 | -14,24 | 0.26 | 0.47 |  |
| After LD I | 20m Sprint | very low | -0,118 | -4,68 | 0.01 | 0.22 |  |
| After LD II | 20m Sprint | very low | -0,021 | -0,84 | -0.08 | 0.13 |  |
| After LD I | 6min Run | very low | 0,401 | 15,58 | -0.51 | -0.30 |  |
| After LD II | 6min Run | very low | 0,354 | 13,82 | -0.46 | -0.25 |  |
| After LD I | Jumping Sideways | very low | 0,003 | 0,11 | -0.11 | 0.10 |  |
| After LD II | Jumping Sideways | very low | 0,052 | 2,07 | -0.16 | 0.05 |  |
| After LD I | Situps | very low | 0,431 | 16,67 | -0.53 | -0.33 |  |
| After LD II | Situps | very low | 0,180 | 7,16 | -0.28 | -0.08 |  |
| After LD I | Standing Broadjump | very low | 0,119 | 4,72 | -0.22 | -0.01 |  |
| After LD II | Standing Broadjump | very low | -0,038 | -1,51 | -0.07 | 0.14 |  |
| After LD I | Pushups | low | -0,477 | -18,34 | 0.37 | 0.58 |  |
| After LD II | Pushups | low | -0,500 | -19,16 | 0.40 | 0.60 |  |
| After LD I | 20m Sprint | low | -0,523 | -19,95 | 0.42 | 0.63 |  |
| After LD II | 20m Sprint | low | -0,138 | -5,51 | 0.03 | 0.24 |  |
| After LD I | 6min Run | low | 0,061 | 2,42 | -0.16 | 0.04 |  |
| After LD II | 6min Run | low | 0,233 | 9,22 | -0.34 | -0.13 |  |
| After LD I | Jumping Sideways | low | -0,247 | -9,77 | 0.14 | 0.35 |  |
| After LD II | Jumping Sideways | low | -0,040 | -1,61 | -0.06 | 0.14 |  |
| After LD I | Situps | low | 0,040 | 1,60 | -0.14 | 0.06 |  |
| After LD II | Situps | low | 0,101 | 4,03 | -0.21 | 0.00 |  |
| After LD I | Standing Broadjump | low | -0,221 | -8,75 | 0.12 | 0.33 |  |
| After LD II | Standing Broadjump | low | -0,036 | -1,45 | -0.07 | 0.14 |  |
| After LD I | Pushups | average | -0,289 | -11,37 | 0.18 | 0.39 |  |
| After LD II | Pushups | average | -0,507 | -19,38 | 0.40 | 0.61 |  |
| After LD I | 20m Sprint | average | -0,185 | -7,34 | 0.08 | 0.29 |  |
| After LD II | 20m Sprint | average | -0,023 | -0,91 | -0.08 | 0.13 |  |
| After LD I | 6min Run | average | 0,320 | 12,54 | -0.42 | -0.22 |  |
| After LD II | 6min Run | average | 0,229 | 11,55 | -0.40 | -0.19 |  |
| After LD I | Jumping Sideways | average | -0,125 | -4,97 | 0.02 | 0.23 |  |
| After LD II | Jumping Sideways | average | 0,060 | 2,39 | -0.16 | 0.04 |  |
| After LD I | Situps | average | 0,166 | 6,15 | -0.26 | -0.05 |  |
| After LD II | Situps | average | -0,057 | -2,28 | -0.05 | 0.16 |  |
| After LD I | Standing Broadjump | average | -0,015 | -0,60 | -0.09 | 0.12 |  |
| After LD II | Standing Broadjump | average | -0,078 | -3,12 | -0.03 | 0.18 |  |
| After LD I | Pushups | high | -0,423 | -16,38 | 0.32 | 0.53 |  |
| After LD II | Pushups | high | -0,489 | -18,75 | 0.38 | 0.59 |  |
| After LD I | 20m Sprint | high | -0,250 | -9,88 | 0.15 | 0.35 |  |
| After LD II | 20m Sprint | high | -0,252 | -9,96 | 0.15 | 0.36 |  |
| After LD I | 6min Run | high | 0,270 | 10,64 | -0.37 | -0.17 |  |
| After LD II | 6min Run | high | 0,083 | 3,29 | -0.19 | 0.02 |  |
| After LD I | Jumping Sideways | high | -0,301 | -11,82 | 0.20 | 0.40 |  |
| After LD II | Jumping Sideways | high | -0,104 | -4,13 | -0.00 | 0.21 |  |
| After LD I | Situps | high | 0,117 | 4,63 | -0.22 | -0.01 |  |
| After LD II | Situps | high | -0,066 | -2,62 | -0.04 | 0.17 |  |
| After LD I | Standing Broadjump | high | -0,177 | -7,01 | 0.07 | 0.28 |  |
| After LD II | Standing Broadjump | high | -0,175 | -6,96 | 0.07 | 0.28 |  |
| After LD I | Pushups | very high | -0,291 | -11,46 | 0.19 | 0.40 |  |
| After LD II | Pushups | very high | -0,398 | -15,48 | 0.29 | 0.50 |  |
| After LD I | 20m Sprint | very high | -0,779 | -28,22 | 0.68 | 0.88 |  |
| After LD II | 20m Sprint | very high | -0,325 | -12,74 | 0.22 | 0.43 |  |
| After LD I | 6min Run | very high | -0,049 | -1,96 | -0.05 | 0.15 |  |
| After LD II | 6min Run | very high | -0,061 | -2,32 | -0.05 | 0.16 |  |
| After LD I | Jumping Sideways | very high | -0,004 | -0,14 | -0.10 | 0.11 |  |
| After LD II | Jumping Sideways | very high | 0,060 | 2,34 | -0.16 | 0.05 |  |
| After LD I | Situps | very high | -0,063 | -2,51 | -0.04 | 0.17 |  |
| After LD II | Situps | very high | -0,150 | -5,84 | 0.04 | 0.25 |  |
| After LD I | Standing Broadjump | very high | -0,321 | -12,59 | 0.22 | 0.43 |  |
| After LD II | Standing Broadjump | very high | -0,219 | -8,66 | 0.11 | 0.32 |  |

SEB = Socioeconomic Background; LD I = first pandemic lockdown, LD II = second pandemic lockdown; Estimates are non-standardized

**Table 4** Effects Time on Body Size and effects of Body Size, Time and their interaction on Motor Performance (overall and per Motor Domain)

| *Outcome* | *Term* | *Est.* | *SE* | *t* | *p* |
| --- | --- | --- | --- | --- | --- |
| *Size* | *Time* | *-0.00* | *0.00* | *-2.59* | *0.01* |
| *20m Run* | *Time* | *-0.00* | *0.03* | *-0.15* | *0.88* |
| *20m Run* | *Size* | *16.35* | *41.59* | *0.39* | *0.69* |
| *20m Run* | *Time x Size* | *-0.01* | *0.02* | *-0.41* | *0.68* |
| *Side Jumps* | *Time* | *0.00* | *0.04* | *0.14* | *0.89* |
| *Side Jumps* | *Size* | *-51.87* | *53.10* | *-0.98* | *0.33* |
| *Side Jumps* | *Time x Size* | *0.02* | *0.03* | *0.95* | *0.34* |
| *Push-ups* | *Time* | *0.02* | *0.04* | *0.51* | *0.61* |
| *Push-ups* | *Size* | *-71.77* | *55.63* | *-1.29* | *0.20* |
| *Push-ups* | *Time x Size* | *0.03* | *0.03* | *1.25* | *0.21* |
| *Sit-ups* | *Time* | *-0.01* | *0.03* | *-0.19* | *0.85* |
| *Sit-ups* | *Size* | *-13.67* | *51.66* | *-0.26* | *0.79* |
| *Sit-ups* | *Time x Size* | *0.01* | *0.03* | *0.24* | *0.81* |
| *Long Jump* | *Time* | *-0.01* | *0.03* | *-0.29* | *0.77* |
| *Long Jump* | *Size* | *-32.71* | *49.72* | *-0.66* | *0.51* |
| *Long Jump* | *Time x Size* | *0.02* | *0.02* | *0.65* | *0.52* |
| *6min Run* | *Time* | *-0.04* | *0.03* | *-1.44* | *0.15* |
| *6min Run* | *Size* | *-41.49* | *40.68* | *-1.02* | *0.31* |
| *6min Run* | *Time x Size* | *0.02* | *0.02* | *0.98* | *0.33* |

Effects of Time (numeric, between 2011 to 2019) on Body Size (‘Size’ in kg, first line), and effects of Time and Size, and their Interaction on motor performance per Motor Domain. Size is given in meters

**Table 5** Additional sensitivity analysis of the effects of Time, SEB, Motor Domain, and their interactions on motor performance after exclusion of 6min run and sit-ups

| *Term* | *F* | *NumDF* | *DenDF* | *p* | *pn²* |
| --- | --- | --- | --- | --- | --- |
| Time | 3.87 | 2 | 33405.76 | 0.02 | <0.01 |
| SEB | 415.43 | 1 | 810.89 | 0.00 | 0.34 |
| Motor Domain | 5790.86 | 5 | 274301.57 | 0.00 | 0.10 |
| Sex | 855.14 | 1 | 66958.34 | 0.00 | 0.01 |
| Time x SEB | 0.16 | 2 | 37915.11 | 0.85 | <0.01 |
| Time x Motor Domain | 103.40 | 10 | 274295.42 | 0.00 | <0.01 |
| SEB x Motor Domain | 22.24 | 5 | 274207.56 | 0.00 | <0.01 |
| Time x SEB x Motor Domain | 6.71 | 10 | 274260.42 | 0.00 | <0.01 |

The 6-minute run and sit-ups showed performance increments during the pandemic relative to pre-pandemic performance levels, thereby obscuring the adverse effects of the pandemic on other motor domains. Consequently, to ascertain the mean negative impact across the remaining four domains, performances in the 6-minute run and sit-ups were omitted from the analysis in the current model

**Table 6** Additional sensitivity analysis of the effects of Time, SEB, Motor Domain, and their interactions on motor performance after exclusion of potential outliers

| *Term* | *F* | *NumDF* | *DenDF* |  | *p* | *pn²* |
| --- | --- | --- | --- | --- | --- | --- |
| Time | 8.95 | 2 | 30334.09 |  | 0.00 | <0.01 |
| SEB | 449.39 | 1 | 812.73 |  | 0.00 | 0.36 |
| Motor Domain | 6675.66 | 5 | 309276.33 |  | 0.00 | 0.10 |
| Sex | 751.58 | 1 | 64556.11 |  | 0.00 | 0.01 |
| Time x SEB | 0.71 | 2 | 34422.49 |  | 0.49 | <0.01 |
| Time x Motor Domain | 114.37 | 10 | 309234.60 |  | 0.00 | <0.01 |
| SEB x Motor Domain | 29.32 | 5 | 309278.60 |  | 0.00 | <0.01 |
| Time x SEB x Motor Domain | 8.86 | 10 | 309306.22 |  | 0.00 | <0.01 |

Outliers were identified and excluded according to the 2SD criterion, whereby all observations exceeding twice the standard deviation from the mean of the sample were removed

**Table 7** Additional sensitivity analysis on linear and quadratic effects of Time (numeric) on motor performance (overall and per Motor Domain)

| Outcome | Term | *Est.* | *SE* | *t* | *p* |
| --- | --- | --- | --- | --- | --- |
| Motor Performance | Time | -0.02 | 0.01 | -2.13 | 0.03 |
| Motor Performance | Time_quad_ | 0.00 | 0.00 | 1.53 | 0.13 |
| 20m Run | Time | -0.02 | 0.01 | -2.13 | 0.03 |
| 20m Run | Time_quad_ | 0.00 | 0.00 | 1.53 | 0.13 |
| Side Jumps | Time | 0.12 | 0.01 | 9.84 | 0.00 |
| Side Jumps | Time_quad_ | -0.01 | 0.00 | -5.96 | 0.00 |
| Push-ups | Time | 0.07 | 0.01 | 5.59 | 0.00 |
| Push-ups | Time_quad_ | 0.00 | 0.00 | 1.02 | 0.31 |
| Sit-ups | Time | -0.09 | 0.01 | -7.24 | 0.00 |
| Sit-ups | Time_quad_ | 0.01 | 0.00 | 6.74 | 0.00 |
| Long Jump | Time | 0.01 | 0.01 | 1.22 | 0.22 |
| Long Jump | Time_quad_ | -0.00 | 0.00 | -0.20 | 0.84 |
| 6min Run | Time | 0.07 | 0.01 | 7.26 | 0.00 |
| 6min Run | Time_quad_ | -0.01 | 0.00 | -8.58 | 0.00 |
|  |  |  |  |  |  |


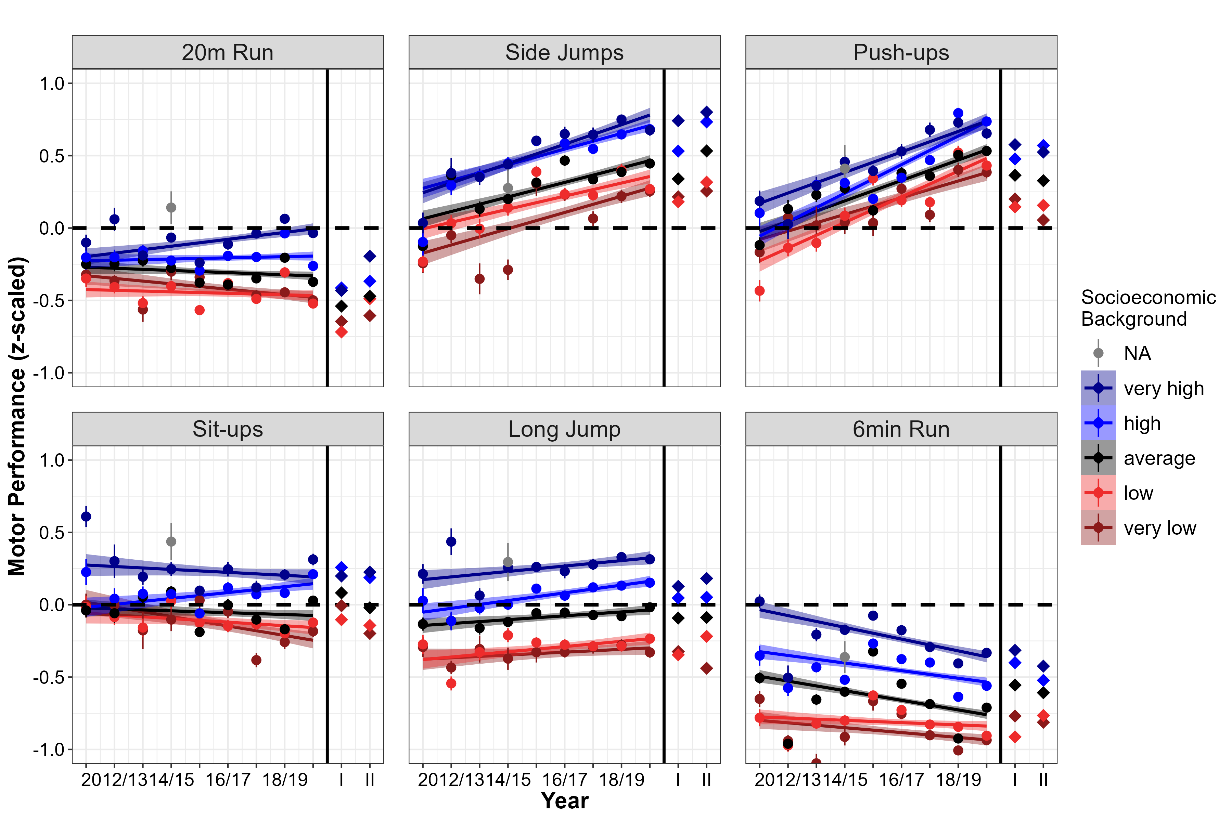


**Figure 1** Girls motor performance per motor domain and school-level socioeconomic background (SEB) before and during the pandemic lockdowns (LD I and LD II). Secular trends of motor performance from 2011 to 2019 (n = 53,570) and during the pandemic (after LD I and LD II, n = 15,426) per Motor Domain (panel) and SEB (color). Average motor performance (z-transformed quantile-normalized percentile values) is displayed on the y-axis, and school years from 2011/12 to 2022/23 are displayed on the x-axis. Linear secular trends (straight lines) are based on 2011/12 to 2019/20 data (points) for each Motor Domain and SEB separately. Motor performance values during the pandemic (rhombs) are shown to the right of the straight vertical black line per panel. Error margins represent standard errors.


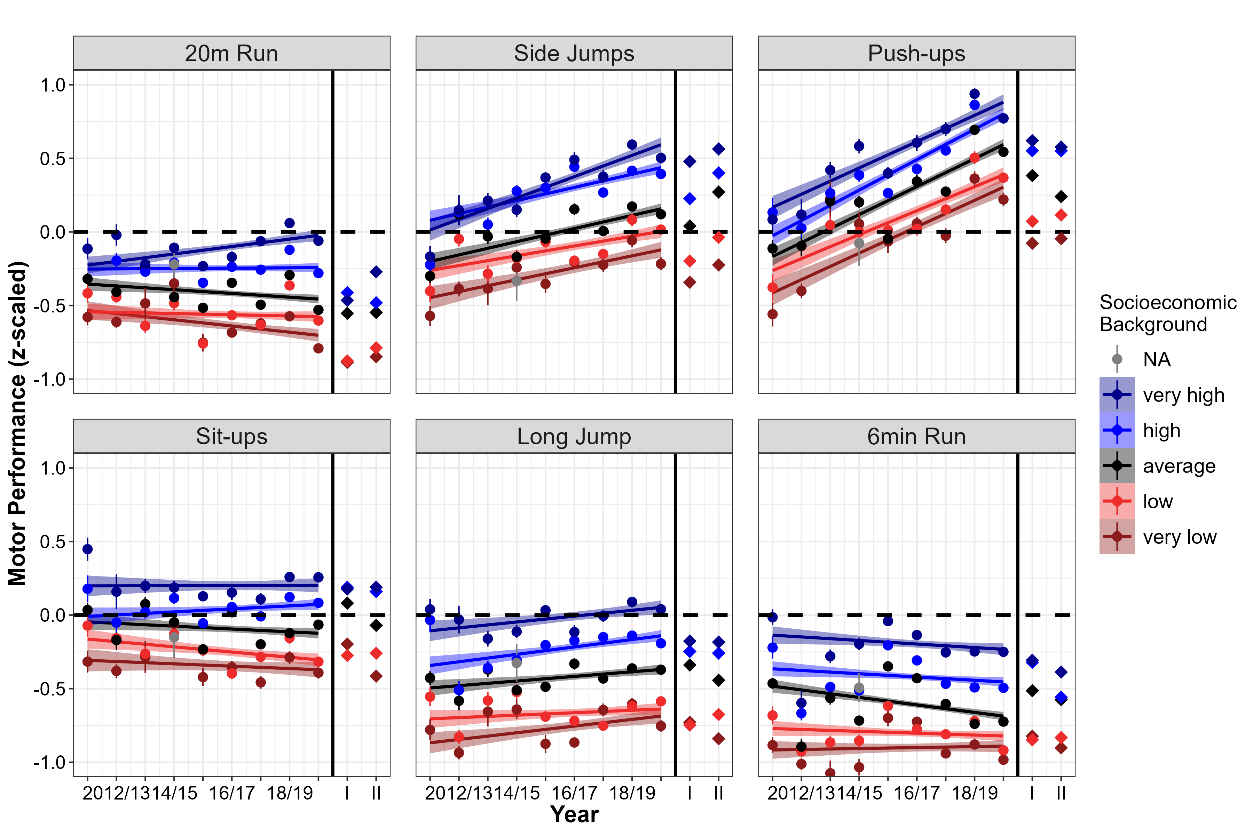


**Figure 2** Boys motor performance per motor domain and school-level socioeconomic background (SEB) before and during the pandemic lockdowns (LD I and LD II). Secular trends of motor performance from 2011 to 2019 (n = 53,570) and during the pandemic (after LD I and LD II, n = 15,426) per Motor Domain (panel) and SEB (color). Average motor performance (z-transformed quantile-normalized percentile values) is displayed on the y-axis, and school years from 2011/12 to 2022/23 are displayed on the x-axis. Linear secular trends (straight lines) are based on 2011/12 to 2019/20 data (points) for each Motor Domain and SEB separately. Motor performance values during the pandemic (rhombs) are shown to the right of the straight vertical black line per panel. Error margins represent standard errors. For boys only.
